# Supplementary material for: Innovations in Snake Venom-Derived Therapeutics: A Systematic Review of Global Patents and Their Pharmacological Applications
Source: Toxins (Basel). 2025 Mar 14;17(3):136. doi: 10.3390/toxins17030136 (PMC11945783; doi:10.3390/toxins17030136)
Supplement: Supplementary file 1 [file toxins-17-00136-s001.zip › toxins-3327235-supplementary.pdf]

Title	Publication number	Access link
Anticoagulant drug based on cobra venom PIII type metalloprotease and applications thereof	CN105567666 (A); CN105567666 (B)	https://worldwide.espacenet.com/publicationDetails/biblio?DB=EPODOC&adjacent=true&locale=en_EP&FT=D&date=20160511&CC=CN&NR=105567666A&KC=A
Anti-IL-4R single-chain antibody and snake venom L-amino acid oxidase fusion protein and application thereof	CN106632686 (A); CN106632686 (B)	https://worldwide.espacenet.com/publicationDetails/biblio?DB=EPODOC&adjacent=true&locale=en_EP&FT=D&date=20170510&CC=CN&NR=106632686A&KC=A
Anti-inflammatory polypeptide DAvp-1 in snake venom and application of anti-inflammatory polypeptide DAvp-1	CN113388020 (A); CN113388020 (B)	https://worldwide.espacenet.com/publicationDetails/biblio?DB=EPODOC&adjacent=true&locale=en_EP&FT=D&date=20210914&CC=CN&NR=113388020A&KC=A
Application of cobra venom in preparation of medicament for treating chronic obstructive pulmonary disease	CN104434981 (A)	https://worldwide.espacenet.com/publicationDetails/biblio?DB=EPODOC&adjacent=true&locale=en_EP&FT=D&date=20150325&CC=CN&NR=104434981A&KC=A
Application of postsynaptic neurotoxin, cardiotoxin, cytotoxin, phospholipase A2 and crude venom of cobra in	CN111617108 (A)	https://worldwide.espacenet.com/publicationDetails/biblio?DB=EPODOC&adjacent=true&locale=en_EP&FT=D&date=20200904&CC=CN&NR=111617108A&KC=A

resisting virus infection		
Application of snake venom polypeptide Hc-CATH in preparation of medicine for preventing and treating Zika virus infection	**CN115957303 (A)**	**https://worldwide.espacenet.com/publicationDetails/biblio?DB=EPODOC&adjacent=true&locale=en_EP&FT=D&date=20230414&CC=CN&NR=115957303A&KC=A**
APPLICATION OF VIPER VENOM HEMOCOAGULASE IN PREPARATION OF DRUG FOR REVERSING ANTICOAGULATION EFFECT OF BLOOD COAGULATION FACTOR XA INHIBITOR	**WO2023168743 (A1)**	**https://worldwide.espacenet.com/publicationDetails/biblio?DB=EPODOC&adjacent=true&locale=en_EP&FT=D&date=20230914&CC=WO&NR=2023168743A1&KC=A1**
COMPOSITION AND METHOD FOR ORAL DELIVERY OF STABLE FORMULATIONS OF COBRA VENOM	**US2019336572 (A1)**	**https://worldwide.espacenet.com/publicationDetails/biblio?DB=EPODOC&adjacent=true&locale=en_EP&FT=D&date=20191107&CC=US&NR=2019336572A1&KC=A1**
Composition for preventing ameliorating or treating arthritis comprising low	**KR20190102909 (A)**	**https://worldwide.espacenet.com/publicationDetails/biblio?DB=EPODOC&adjacent=true&locale=en_EP&FT=D&date=20190904&CC=KR&NR=20190102909A&KC=A**

molecular weight peptide isolated from heat-treated snake venom as effective component		
Composition For Preventing Or Treating Rheumatoid Arthritis, Comprising Snake Venom	US2022362356 (A1)	https://worldwide.espacenet.com/publicationDetails/biblio?DB=EPODOC&adjacent=true&locale=en_EP&FT=D&date=20221117&CC=US&NR=2022362356A1&KC=A1
Composition of anti-venom of the Korean salmosa species' venom	KR20220170290 (A)	https://worldwide.espacenet.com/publicationDetails/biblio?DB=EPODOC&adjacent=true&locale=en_EP&FT=D&date=20221229&CC=KR&NR=20220170290A&KC=A
Crotoxin Administration for Cancer Treatment	US2015110770 (A1); US9345751 (B2)	https://worldwide.espacenet.com/publicationDetails/biblio?DB=EPODOC&adjacent=true&locale=en_EP&FT=D&date=20150423&CC=US&NR=2015110770A1&KC=A1
Fibrinolytic enzyme from gloydius intermedius venom and preparation method and application thereof	CN110724678 (A); CN110724678 (B)	https://worldwide.espacenet.com/publicationDetails/biblio?DB=EPODOC&adjacent=true&locale=en_EP&FT=D&date=20200124&CC=CN&NR=110724678A&KC=A
Glutamic acid stabilizer for snake venom enzyme preparation and preparation method	CN108273067 (A); CN108273067 (B)	https://worldwide.espacenet.com/publicationDetails/biblio?DB=EPODOC&adjacent=true&locale=en_EP&FT=D&date=20180713&CC=CN&NR=108273067A&KC=A
Immobilized snake venom blood coagulation factor activator and	CN108743924 (A); CN108743924 (B)	https://worldwide.espacenet.com/publicationDetails/biblio?DB=EPODOC&adjacent=true&locale=en_EP&FT=D&date=20181106&CC=CN&NR=108743924A&KC=A

preparation method of activated blood coagulation factor		
Light-operated snake venom polypeptide zinc nano preparation and preparation method and application thereof	**CN105497873 (A); CN105497873 (B)**	**https://worldwide.espacenet.com/publicationDetails/biblio?DB=EPODOC&adjacent=true&locale=en_EP&FT=D&date=20160420&CC=CN&NR=105497873A&KC=A**
Method for extracting blood coagulation factor X activating agent from snake venom	**CN109943554 (A); CN109943554 (B)**	**https://worldwide.espacenet.com/publicationDetails/biblio?DB=EPODOC&adjacent=true&locale=en_EP&FT=D&date=20190628&CC=CN&NR=109943554A&KC=A**
Method for preparing heterodimeric snake venom protein	**NZ753297 (A)**	**https://worldwide.espacenet.com/publicationDetails/biblio?DB=EPODOC&adjacent=true&locale=en_EP&FT=D&date=20211126&CC=NZ&NR=753297A&KC=A**
Method for purifying cobra venom and its products	**CN109929020 (A)**	**https://worldwide.espacenet.com/publicationDetails/biblio?DB=EPODOC&adjacent=true&locale=en_EP&FT=D&date=20190625&CC=CN&NR=109929020A&KC=A**
Modified cobra venom and its physical modification methods and uses	**KR20190007161 (A)**	**https://worldwide.espacenet.com/publicationDetails/biblio?DB=EPODOC&adjacent=true&locale=en_EP&FT=D&date=20190122&CC=KR&NR=20190007161A&KC=A**
Pharmaceutical composition of 4-aminoquinoline derivative and snake venom cytotoxin-CTX1	**CN107737333 (A); CN107737333 (B)**	**https://worldwide.espacenet.com/publicationDetails/biblio?DB=EPODOC&adjacent=true&locale=en_EP&FT=D&date=20180227&CC=CN&NR=107737333A&KC=A**

Pharmaceutical composition of Siramesine and snake venom cytotoxin-CTX1	CN107929717 (A); CN107929717 (B)	https://worldwide.espacenet.com/publicationDetails/biblio?DB=EPODOC&adjacent=true&locale=en_EP&FT=D&date=20180420&CC=CN&NR=107929717A&KC=A
Preparation method of prosperohead agkistrodon halys venom C-type lectin-like protein and application thereof as anticoagulant drug	CN115594746 (A)	https://worldwide.espacenet.com/publicationDetails/biblio?DB=EPODOC&adjacent=true&locale=en_EP&FT=D&date=20230113&CC=CN&NR=115594746A&KC=A
Preparation process for extracting cobratide and snake venom substance A from snake venom	CN117756907 (A)	https://worldwide.espacenet.com/publicationDetails/biblio?DB=EPODOC&adjacent=true&locale=en_EP&FT=D&date=20240326&CC=CN&NR=117756907A&KC=A
Purification preparation method and application of cobra venom CTX (cytotoxin)-4N	CN107098956 (A); CN107098956 (B)	https://worldwide.espacenet.com/publicationDetails/biblio?DB=EPODOC&adjacent=true&locale=en_EP&FT=D&date=20170829&CC=CN&NR=107098956A&KC=A
SNAKE VENOM C FRAGMENT POLYPEPTIDE DERIVATIVE	WO2017190263 (A1)	https://worldwide.espacenet.com/publicationDetails/biblio?DB=EPODOC&adjacent=true&locale=en_EP&FT=D&date=20171109&CC=WO&NR=2017190263A1&KC=A1
Snake venom neurotoxin and application	CN114409757 (A); CN114409757 (B)	https://worldwide.espacenet.com/publicationDetails/biblio?DB=EPODOC&adjacent=true&locale=en_EP&FT=D&date=20220429&CC=CN&NR=114409757A&KC=A
Snake venom polypeptide and	CN117343131 (A)	https://worldwide.espacenet.com/publicationDetails/biblio?DB=EPODOC&adjacent=true&locale=en_EP&FT=D&date=20240105&CC=CN&NR=117343131A&KC=A

application thereof		
Thrombolytic drug based on cobra venom PIIII type metalloproteinase and application of thrombolytic drug	CN105861476 (A); CN105861476 (B)	https://worldwide.espacenet.com/publicationDetails/biblio?DB=EPODOC&adjacent=true&locale=en_EP&FT=D&date=20170511&CC=US&NR=2017128544A1&KC=A1
Uses of Humanized Cobra Venom Factor for Reducing or Preventing Immunogenicity	US2017128544 (A1)	https://worldwide.espacenet.com/publicationDetails/biblio?DB=EPODOC&adjacent=true&locale=en_EP&FT=D&date=20170511&CC=US&NR=2017128544A1&KC=A1
